# Focal scintigraphic findings in clinically suspected tibial stress fractures

**DOI:** 10.1590/0100-3984.2018.51.5e2

**Published:** 2018

**Authors:** Claudio Tinoco Mesquita, Gustavo do Vale Gomes

**Affiliations:** 1 Hospital Universitário Antônio Pedro, Universidade Federal Fluminense, Niterói, RJ, Brazil. E-mail: claudiotinocomesquita@id.uff.br.; 2 Núcleos, Radiologia e Medicina Nuclear, Brasília, DF, Brazil.

Stress fractures are a common clinical problem, especially among soldiers and athletes. At the 2016 Olympics in Rio de Janeiro, stress fractures were detected in 36% of the athletes with abnormal imaging tests, the most common location being the tibia^(1)^. Biomechanical models of stress study during exercise demonstrate that the tibia is the bone subjected to the greatest compressive load during running, as well as that thigh muscle fatigue and the addition of load during exercise significantly increase the risk of developing fractures of stress^(2)^.

The clinical diagnosis of stress fracture can represent a clinical challenge and merits adequate evaluation by imaging methods^(3)^. Planar X-ray and ultrasound, despite their low cost, show limited sensitivity in this context. An abnormal X-ray has a high diagnostic capacity, although it is often a late finding and is uncommon^(3)^. Computed tomography (CT) can add useful anatomical information, but it is not part of the routine investigation of suspected cases of stress fractures. A study conducted by Groves et al. demonstrated that the sensitivity of CT is lower than is that of bone scintigraphy, and that CT can be a better option when scintigraphy produces inconclusive results^(4)^. Recently, the American College of Radiology published the appropriateness criteria for the investigation of patients with suspected stress fractures and recommended the use of magnetic resonance imaging (MRI) as the method of choice for the identification of stress fractures that were not readily diagnosed by conventional radiology^(5)^. MRI offers excellent spatial resolution and superior ability to assess bone marrow involvement. Three-phase bone scintigraphy is considered a useful, effective method for evaluating patients with suspected stress fractures, especially those with lower limb injuries, having the advantages of high sensitivity, wide availability, and low cost^(5)^. However, scintigraphy has limitations due to the possibility of false-positive results in cases of focal infections or small tumors. Novel techniques of bone scintigraphy optimization, such as single-photon emission CT/CT (SPECT/CT), significantly increase the specificity of this method for the detection of stress fractures^(6)^. However, there are no direct studies comparing its accuracy with that of MRI in this context. Therefore, the use of imaging techniques for the diagnostic evaluation of fractures presents an array of evidence that guides clinical use. In contrast, there is a relative scarcity of data in the literature regarding the use of imaging in monitoring the recovery of stress fractures. Classifications of the degree of impairment seen on MRI, such as that proposed Fredericson et al.^(7)^, are widely used but have not been objectively studied for their correlation with patient recovery time. Although quite relevant, defining the optimal recovery time for a given stress fracture continues to pose a challenge for specialists in the area.

In view of the difficulties discussed above, we congratulate Castropil et al. on their study, published in the previous issue of the **Radiologia Brasileira**, which evaluated the potential of scintigraphy to facilitate the clinical management of patients diagnosed with stress fractures^(8)^. Using the score devised by Chisin et al.^(9)^ as the basis for quantification of the findings, the authors demonstrated an objective correlation to estimate the appropriate recovery time after a tibial stress fracture. Castropil et al.^(8)^ found a statistically significant linear relationship between the quantification of uptake and the time required for adequate recovery. These encouraging results, because of the potential impact on the more assertive behavior of patients recovering from stress fractures, open space for new clinical studies. A validation of the relationship found in the study in question, with larger cohorts and stress fractures from other locations, is highly desirable. Other promising analyses could be related to the increase in improvements already available in three-phase bone scintigraphy, such as lateral projections or SPECT of the balance images and quantifications using the SPECT and SPECT/CT images. It is known that CT findings, such as characterization of the periosteal reaction and absence of the fracture line, can be quite useful in evaluating the healing of stress fractures. Therefore, the addition of these anatomical aspects to the greater sensitivity of the metabolic information provided by bone scintigraphy with SPECT/CT could enhance the ability of the method to estimate the time needed for adequate recovery of patients with stress fractures. In conclusion, the authors have provided us with relevant information about the prognostic capacity of bone scintigraphy and have laid the groundwork for subsequent clinical applications.
